# Refugees, Trauma, and Positive Psychological Change: Mindfulness as a Moderator for Posttraumatic Growth

**DOI:** 10.3390/healthcare14030379

**Published:** 2026-02-03

**Authors:** Ertan Yılmaz, Ufuk Bal, Emre Dirican

**Affiliations:** 1 Department of Psychiatry, Faculty of Medicine, Hatay Mustafa Kemal University, Hatay 31000, Turkey; 2 Independent Researcher, Adana 01150, Turkey; ufukbala@hotmail.com; 3 Department of Biostatistics, Faculty of Medicine, Hatay Mustafa Kemal University, Hatay 31000, Turkey; emredir44@hotmail.com

**Keywords:** PTSD, mindfulness, trauma, Refugee, PTG

## Abstract

**Background/Objectives:** Traumatic experiences may lead to both negative and positive outcomes. Positive psychological changes following trauma are commonly referred to as posttraumatic growth (PTG). The present study aims to examine factors associated with posttraumatic growth among Syrian refugees who have been living in Turkey for an extended period. **Methods:** This cross-sectional study included a sample of 240 Syrian refugees. Participants completed the Posttraumatic Stress Disorder Checklist (PCL-5), the Posttraumatic Growth Inventory (PTGI), and the Mindful Attention Awareness Scale (MAAS). Path analysis was conducted to examine the effects of PTSD symptoms and mindfulness levels on posttraumatic growth. In addition, Multivariate Adaptive Regression Spline (MARS) analysis was used to identify threshold values for the contributions of these variables to posttraumatic growth. **Results**: The mean age of the participants was 36.9 ± 10.4 years, and 47% were female. The direct effect of PTSD symptoms on posttraumatic growth was negative and statistically significant (β = −0.291, *p* < 0.001). PTSD symptoms also had an indirect effect on posttraumatic growth through mindfulness (β = −0.254), resulting in a total effect of −0.545. According to the MARS model, when MAAS scores exceeded 78, mindfulness demonstrated a positive effect on posttraumatic growth. **Conclusions:** The findings indicate that PTSD symptoms among refugees are associated with posttraumatic growth through both direct and indirect pathways. Furthermore, mindfulness emerges as a key factor in understanding the development of posttraumatic growth in this population.

## 1. Introduction

Refugee experiences are often accompanied by multiple adverse and traumatic life events, ranging from displacement and poverty to exposure to torture. The civil war that began in Syria in 2011 triggered a major humanitarian crisis, forcing millions of individuals to flee their country. As of 2023, Turkey hosts approximately 3 million Syrian refugees [1].

This population has been granted temporary protection status in Turkey. However, for consistency with the existing literature, the term refugee is used throughout this article. Mental health disorders are highly prevalent among refugees and asylum seekers, with posttraumatic stress disorder (PTSD) being one of the most commonly reported and extensively studied conditions in this population [2].

The prevalence of PTSD among refugees varies considerably across studies [3]. A meta-analysis that accounted for methodological differences reported that approximately one-third of refugees meet the criteria for PTSD [4]. Similarly, a meta-analysis conducted by Nguyen et al. found a PTSD prevalence of 31% among Syrian refugees resettled in Western countries [5].

Although the research has traditionally focused on the negative psychological consequences of exposure to traumatic and adverse life events, growing evidence suggests that such experiences do not inevitably result in negative outcomes [6]. While early studies used different terms to describe positive psychological changes following trauma, the concept of posttraumatic growth (PTG) has gained broad acceptance in the more recent literature. PTG was defined by Tedeschi and Calhoun as the experience of positive psychological change resulting from the struggle with highly challenging life circumstances [7].

Posttraumatic growth encompasses five distinct domains: Relating to Others, Appreciation of Life, Personal Strength, New Possibilities, and Spiritual Change. Evidence indicates that posttraumatic growth has been observed at varying levels across different refugee populations [8,9,10]. These findings suggest that posttraumatic growth is not confined to a specific cultural context but may represent a more universal psychological experience.

The presence of posttraumatic growth does not exclude the coexistence of negative psychological outcomes, particularly posttraumatic stress disorder (PTSD) [11]. Research examining the relationship between posttraumatic growth and PTSD consistently suggests that these phenomena are distinct constructs that can coexist rather than representing opposite ends of a single continuum [12].

A range of factors contributing to the development of PTG has been identified in the literature, including optimism [13], problem-oriented coping strategies [14], religiosity [15], perceived social support [16], and self-compassion [17]. Another factor that has been suggested to play a role in posttraumatic growth is mindfulness. Mindfulness is commonly conceptualized as a state of awareness characterized by nonjudgmental, moment-to-moment attention to present experiences, accompanied by openness and nonreactivity [18].

Bishop and colleagues proposed a two-component model of mindfulness. The first component involves the self-regulation of attention, whereby attention is directed toward present-moment experiences, facilitating greater awareness of thoughts, emotions, and bodily sensations. The second component refers to the adoption of a particular orientation toward one’s experiences in the present moment, characterized by curiosity, openness, and acceptance [19]. Dispositional mindfulness, by contrast, refers to an individual’s inherent tendency to demonstrate these aspects of mindful awareness in daily life [20].

There is considerable evidence that mindfulness influences responses to trauma. For example, Boelen et al. reported that individuals’ levels of mindfulness predicted subsequent PTSD symptoms [21]. Similarly, Im et al. identified a negative association between mindfulness and trauma-related symptomatology in a sample of university students [22]. In addition, a meta-analysis conducted by Liu et al. demonstrated that Mindfulness-Based Stress Reduction (MBSR) interventions were effective in reducing PTSD symptoms [23].

There is also a growing number of studies on the relationship between mindfulness and PTG. Research involving cancer patients has shown that MBSR interventions are associated with increased levels of PTG [24,25]. In a study of firefighters, Huang et al. reported that dispositional mindfulness was negatively associated with PTSD and positively associated with posttraumatic growth [26]. Similarly, Wen et al. found a positive relationship between mindfulness and posttraumatic growth, alongside a negative relationship between mindfulness and PTSD, in a sample of aid workers [27].

In contrast, other studies have reported opposing findings. For example, Liu et al. found that dispositional mindfulness negatively predicted posttraumatic growth in a study of cancer patients [28]. These findings highlight the complexity and context-dependent nature of the relationship between mindfulness and posttraumatic growth.

Refugees are a special group exposed to different traumatic experiences and life difficulties before, during, and after migration. Levels of PTG and its associated factors may therefore differ across trauma-exposed groups. Despite this, empirical evidence on posttraumatic growth and its correlates among refugees living in developing countries, such as Turkey, remains limited. Therefore, the primary aim of the present study is to contribute to a clearer understanding of the pathways leading to PTG in refugee populations. Specifically, the first objective is to examine sociodemographic factors associated with posttraumatic growth among Syrian refugees residing in the southern region of Turkey. The second objective is to investigate the mediating role of mindfulness in the relationship between trauma-related factors and posttraumatic growth. In addition, the mediating role of mindfulness will be examined across the five domains of posttraumatic growth.

## 2. Materials and Methods

### 2.1. Sample and Design

The study was conducted in Adana, a city in the southern region of Turkey with a high concentration of Syrian refugees, between January 2023 and January 2024. According to official records, Adana ranks as the fourth city hosting the largest number of Syrian refugees, with an estimated population of nearly 200,000 individuals. At the time of data collection, more than 3.5 million Syrians were residing in Turkey [1].

Due to the temporary protection status granted in Turkey, high population mobility among Syrians made it difficult to define a fixed sampling frame. Therefore, a snowball sampling method was employed. Five neighborhoods with high concentrations of Syrian refugees were initially identified. Residential addresses were determined through contact with neighborhood heads and local community leaders. To reach individuals living outside these neighborhoods, Syrian refugees who sought services from healthcare institutions and interpreters working in hospitals were also contacted. One participant was interviewed from each household. In cases where no eligible participant could be reached or participation was declined, the next household on the list was approached. Interviews were conducted by trained interpreters who were fluent in Turkish and native speakers of Arabic, all of whom had at least a high school level of education. All questionnaires were administered in Arabic.

All interviewers received training prior to the commencement of the study. The training covered the study objectives, ethical considerations, and the content of the survey instruments, with particular emphasis on accurate and complete administration of the questionnaires. In addition, the clarity of the survey items and potential areas of difficulty for participants were discussed. The surveys were primarily self-administered by participants. However, in cases where participants had difficulty understanding the items or were illiterate, interviewers provided assistance to ensure accurate completion.

The study protocol was approved by the Ethics Committee of Adana City Training and Research Hospital (29.07.2020-62). In addition, the required administrative permissions were obtained from the Adana Provincial Directorate of Migration Management, operating under the General Directorate of Migration Management of the Ministry of Interior.

### 2.2. Inclusion Criteria

1. Being over 18 years old.
2. Being a Syrian refugee.
3. Having the cognitive capacity to understand and answer the questionnaire.
4. Having volunteered to participate in the study.

### 2.3. Research Instruments

#### 2.3.1. Sociodemographic Form

A sociodemographic questionnaire was developed by the researchers, informed by previous studies conducted with refugee populations. The form collected information on participants’ age, gender, marital status, number of children, years of education, employment status, and income level. In addition, questions assessed Turkish-language proficiency, duration of refugee status, living arrangements (living alone or with family), household size, the presence of relatives remaining in Syria, and receipt of regular assistance.

#### 2.3.2. Posttraumatic Stress Disorder Checklist for the Diagnostic and Statistical Manual of Mental Disorders, Fifth Edition (PCL-5)

The Posttraumatic Stress Disorder Checklist for the Diagnostic and Statistical Manual of Mental Disorders, Fifth Edition (PCL-5) is a 20-item self-report measure designed to assess symptoms of PTSD. The items are grouped into four symptom clusters: re-experiencing, avoidance, negative alterations in cognition and mood, and hyperarousal. Each item is rated on a 5-point Likert scale ranging from 0 to 4, yielding a total score between 0 and 80 [29]. The scale has been translated into Arabic and validated, demonstrating high internal consistency (Cronbach’s α = 0.85). A cut-off score of 23 has been shown to predict PTSD with a sensitivity of 82% and a specificity of 70% [30].

#### 2.3.3. Posttraumatic Growth Inventory (PTGI)

The Posttraumatic Growth Inventory (PTGI) was developed to assess positive psychological changes reported by individuals following exposure to traumatic events. The inventory consists of 21 items encompassing five subscales: New Possibilities, Relating to Others, Personal Strength, Spiritual Change, and Appreciation of Life. Items are rated on a 6-point Likert scale ranging from 0 (“I did not experience this change as a result of the crisis”) to 5 (“I experienced this change to a very great degree as a result of the crisis”). Higher total scores reflect greater levels of positive psychological change following adverse life experiences. The PTGI was translated into Arabic and subsequently back-translated into English in two independent studies. Rizkalla et al. reported good internal consistency for the total scale (Cronbach’s α = 0.89), with subscale reliability coefficients ranging from 0.47 (Appreciation of Life) to 0.83 (Relating to Others) [31]. In addition, Kira et al. demonstrated that the 21-item Arabic version of the PTGI exhibited excellent internal consistency (Cronbach’s α = 0.96) [32].

#### 2.3.4. Mindful Attention Awareness Scale (MAAS)

The MAAS is a 15-item self-report scale to assess dispositional (or personality trait) awareness. The MAAS, developed by Brown and Ryan, measures the tendency toward awareness or inattention using a 6-point Likert scale with higher scores indicating greater levels of mindfulness [20]. Confirmatory factor analysis conducted on a sample of university students supported the scale’s unidimensional factor structure, and the original version demonstrated good internal consistency (Cronbach’s α = 0.82). The Arabic version of the MAAS was translated and validated by Rayan et al., who reported high internal consistency for the scale (Cronbach’s α = 0.92) [33].

### 2.4. Statistics

All analyses were performed using SPSS 27.0 (IBM Corp., Armonk, NY, USA) and R Studio (v2023.06.1 + 524) software. To examine the direct and indirect effects between scales, a path model was constructed using IBM SPSS AMOS 24 (Analysis of Moment Structures) software, and evaluations were made based on this model. Additionally, to model the dependent variable PTG, the Multivariate Adaptive Regression Spline (MARS) method was applied, which offers a more flexible approach for capturing non-linear relationships; analyses were performed in the R environment (packages with earth v7.0-1, caret v5.3.3).

Descriptive statistics were reported as mean ± standard deviation for continuous variables, median (q1–q3) when data were not normally distributed, and frequency and percentage for categorical variables. The normality of continuous variables was assessed using the Shapiro–Wilk and Kolmogorov–Smirnov tests. For comparisons between two groups, Student’s *t*-test was used for variables meeting the assumption of normal distribution, and the Mann–Whitney U test was used when this assumption was not met. A significance level of *p* < 0.05 was accepted for all tests.

The fit of the established path model was assessed using multiple goodness-of-fit indices, including χ^2^/df, RMSEA, CFI, TLI, and SRMR. Criteria for acceptable fit based on CMIN/df (χ^2^/df) are as follows: ≤2 → excellent fit; ≤3 → good fit; ≤5 → acceptable fit; RMSEA < 0.08; CFI > 0.90; GFI between 0.90 and 0.95 → acceptable fit; and SRMR < 0.08. Mediation analyses were conducted using structural equation modeling. Indirect effects were evaluated using a bootstrap procedure with 5000 resamples, and bias-corrected bootstrap confidence intervals were computed. Statistical significance of indirect effects was determined based on whether the confidence interval excluded zero. Because the mediation model was just-identified, global fit indices were not interpreted. All parameters were estimated using the Maximum Likelihood (ML) method in AMOS.

Finally, to address model adequacy and minimize overfitting, the MARS model was fitted using the original observed dataset (*N* = 240) only. Model performance was evaluated using 10-fold cross-validation, and model complexity was reported based on the number of basis functions retained in the final model. To assess the robustness of the estimated knot locations, a bootstrap procedure was applied, and the stability of knot placement across resamples was examined. All reported MARS results and inferences are therefore based exclusively on analyses conducted on the observed data. The performance of the MARS model created for the dependent variable PTG was evaluated using measures such as R^2^**,** Generalized Cross-Validation (GCV), and goodness-of-fit R-squared (GRSq).

## 3. Results

A total of 240 participants were included in the study, 47% of whom were female. The mean age of the sample was 36.9 ± 10.4 years. Most participants were married (88.3%) and lived with their families (96.7%), with an average household size of approximately five individuals. The mean duration of education was 6.5 ± 5.3 years. Nearly half of the participants reported having relatives residing in Syria (51.7%), and 55.5% were employed in regular jobs. Additional sociodemographic characteristics are presented in Table 1.

The mean PTG score of the participants was 67.9 ± 14.7. Female participants reported significantly higher levels of PTG compared to males (*p* = 0.001). No significant gender differences were observed for PTSD symptoms (*p* = 0.688) or mindfulness (*p* = 0.29). Comparisons of key variables indicated that income (*p* < 0.001) and gender (*p* = 0.011) were significantly associated with PTG, which was therefore treated as the dependent variable in subsequent analyses (Table 2). In addition, PTG was negatively correlated with age (*r* = −0.219; *p* < 0.001) and positively correlated with duration of education (*r* = 0.317; *p* < 0.001).

**Figure 1:**
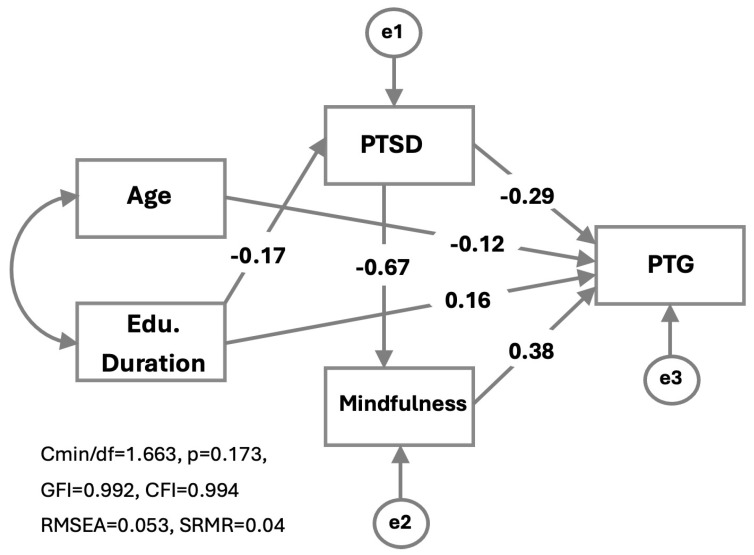
Pathways significantly associated with PTG with standardized weights. Single-headed arrows indicate hypothesized directional (regression) effects, whereas double-headed arrows represent covariances between variables. All paths displayed in the figure correspond to the predefined study hypotheses. Although age and education duration were allowed to covary in the model, this association was not included in the figure because it was not part of the hypothesized relationships. The terms e1, e2, and e3 denote error terms associated with the endogenous variables. Standardized path coefficients are shown.

PTG was included in the path model alongside PTSD and mindfulness, with mindfulness specified as a mediating variable. Age and duration of education were also incorporated as covariates in the model. Following the removal of non-significant paths, the model was refined to its final form, as illustrated in Figure 1. The final model demonstrated a good fit to the data (CMIN/df = 1.663; *p* = 0.173; GFI = 0.992; CFI = 0.994; RMSEA = 0.053).

The direct effect of PTSD symptoms on PTG was negative and statistically significant (β = −0.291; *p* < 0.001). In addition, PTSD showed a significant indirect effect on posttraumatic growth through mindfulness (indirect effect = −0.254), resulting in a total effect of −0.545. These findings indicate that higher levels of PTSD are associated with lower levels of PTG through both direct and indirect pathways.

Mindfulness had a positive and significant direct effect on PTG (β = 0.378; *p* < 0.001), suggesting that higher levels of mindfulness are associated with greater PTG. Furthermore, PTSD symptoms had a strong and negative effect on mindfulness (β = −0.674; *p* < 0.001), which accounts for the observed indirect effect of PTSD on PTG.

Duration of education showed significant effects on both PTSD (β = −0.165; *p* = 0.01) and PTG (β = 0.163; *p* < 0.001). When the indirect effect of education duration on PTG (0.05) was taken into account, the total effect increased to 0.253. Although the direct effect of age on PTG was relatively small, it was statistically significant (β = −0.118; *p* = 0.015) (Table 3).

**Figure 2:**
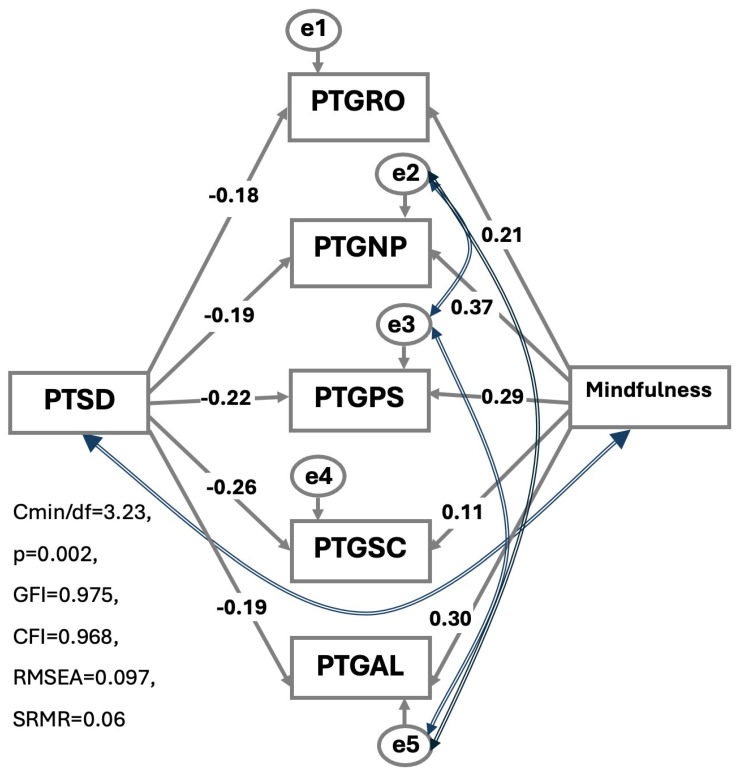
MAAS and PCL scale pathways for PTG subscales. Residual covariances were added between PTG New Possibilities (NP) and PTG Relating Others (RO), PTG Personal Strength (PS) and PTG Appreciation of Life (AL), and PTG Spiritual Change (SP) as these subscales reflect overlapping aspects of posttraumatic growth experiences. This specification was theoretically justified and improved the overall model fit. Cronbach’s alpha values were RO = 0.80, NP = 0.72, PS = 0.63, and AL = 0.76. For the two-item SC sub-scale inter-item correlation was r = 0.20.

Figure 2 shows the effects of PTSD and mindfulness on the PTG subdimensions. Among these dimensions, the strongest negative effect of PTSD was observed for the Spiritual Change domain (β = −0.263; *p* = 0.001), indicating that higher levels of PTSD were associated with significantly lower levels of Spiritual Change. In contrast, the weakest negative association was found for the Relating to Others dimension (β = −0.179; *p* = 0.028). The negative effect of PTSD on the Personal Strength domain was comparatively more limited.

Mindfulness has the strongest effect on the New Possibilities subdomain of PTG (β = 0.372; *p* < 0.001). Higher levels of mindfulness were also significantly associated with greater scores in the Relating to Others, Personal Strength, and Appreciation of Life domains (β = 0.214 with *p* = 0.009, β = 0.292 with *p* < 0.001, and β = 0.303 with *p* < 0.001, respectively). In contrast, mindfulness did not have a statistically significant effect on the Spiritual Change domain (β = 0.111; *p* = 0.176) (Table 4).

We constructed a MARS model with variables that we thought could be effective in PTG scores, in addition to PTSD and mindfulness scores (age, gender, education duration, income). Female gender showed a higher association with PTG; women scored, on average, approximately 5.3 points higher than men. In addition, income level above a specified threshold was associated with an increase of approximately 12.6 points in PTG, and these effects were linear and constant within the model. The relationship between PTSD and PTG exhibited a threshold-based pattern: when PTSD scores exceeded 8, each one-unit increase corresponded to an approximate 0.75-point decrease in PTG. However, when PTSD scores were above 22, the direction of the association reversed, with each additional unit corresponding to an increase of approximately 0.66 points in PTG, suggesting different dynamics at moderate and high levels of PTSD. The association between mindfulness and PTG also followed a two-stage structure. No significant effect was observed for mindfulness scores below 58, whereas increases in mindfulness between 58 and 78 were associated with an approximate 2.2-point decrease in PTG per unit. In contrast, when mindfulness scores exceeded 78, the inclusion of an additional positive basis function led to a reversal of the slope, indicating that further increases in mindfulness contributed positively to PTG (net slope ≈ +0.9 points per unit). Finally, years of education demonstrated a threshold effect; more than 10 years of education was associated with an increase of approximately 1.15 points in PTG for each additional year.

PTG = 105.5373
+5.258996 × GenderFemale
+12.63022 × İncome > 126$ (Min. Wage)
−0.7490192 × max (0, PTSD–8)
+0.6600576 × max (0, PTSD–22)
−2.206769 × max (0, Mindfulness–58)
+3.115925 × max (0, Mindfulness–78)
+1.151465 × max (0, Edu_duration–10)

## 4. Discussion

A central question guiding the present study was whether psychopathology is an inevitable outcome for refugee populations following exposure to traumatic and adverse experiences or whether positive psychological changes may also emerge. The findings of this study provide evidence for the presence of posttraumatic growth among refugees. These results suggest that, alongside psychological distress, refugees may also experience meaningful positive psychological changes following trauma.

The mean PTG scores observed in the present study ranged from moderate to high levels. These findings are consistent with those reported by Özdemir et al. [34]. Moreover, when compared with other studies conducted among Syrian refugee populations, the PTG level identified in this study appears to be relatively higher, though still within a moderate range [15,31,35].

In our study, significantly higher PTG values were found in women compared to men. Conversely, no gender difference was found in PTSD. Our findings are consistent with a meta-analysis conducted by Vishnevsky et al. [36]. Furthermore, while some studies found a significant relationship between PTG and gender [12,37], no significant relationship was found in other studies [34,35,38,39,40,41]. Women and men being exposed to different types of trauma may affect PTG development to varying degrees. Another possible explanation is that women may be more willing to share their negative experiences, consequently triggering cognitive processes.

Age has been identified as one of the factors associated with PTG. In the present study, age showed a statistically significant but relatively small effect on posttraumatic growth. This finding is consistent with previous studies reporting a negative association between age and posttraumatic growth [42,43,44]. However, other studies have found no significant relationship between age and posttraumatic growth [31,34,39,40,41], suggesting that the role of age in PTG may vary across populations and contexts. Greater cognitive flexibility in the younger age group compared to the older group may facilitate PTG development. Additionally, the older group’s greater exposure to negative life events may have a negative impact on PTG.

Another important finding of our study is that the duration of education has a direct positive effect on PTG, as well as an indirect effect by reducing PTSD levels, thereby increasing PTG. The positive effect of education level on PTG has been supported by previous studies [15,35,39]. However, there are also studies that did not find a correlation between education and PTG [31,34]. These results suggest that education can affect growth in two different ways.

One possible explanation is that individuals with higher levels of education may have access to a broader range of coping strategies when dealing with stress. Higher educational attainment may also confer advantages related to employment opportunities and income stability. Conversely, it has been suggested that individuals with lower levels of education may rely more heavily on interpersonal relationships as an alternative coping resource, potentially fostering growth in relational domains [45,46].

While some studies have reported a positive association between income level and PTG [31,43], Ersahin et al. found no significant relationship between income and PTG [15]. In our study, higher income levels were associated with greater PTG. In addition, mindfulness levels were significantly higher among participants with higher income. These findings suggest that income level may influence PTG both directly and indirectly, potentially through its association with mindfulness.

PTSD scores had a significantly direct negative effect on PTG. Furthermore, PTSD strongly negatively affected PTG indirectly through mindfulness. However, according to our advanced analyses, the relationship between PTSD and PTG exhibited a threshold-based pattern. Mild and moderate PTSD levels had a negative effect on PTG. Conversely, an increase in PTSD levels (above 22) had a positive effect on PTG. Another important finding of our study is that PTSD had a negative effect on all subscales of PTG, but the strongest effect size was observed in the Spiritual Change subscale. Rizkalla et al. found no relationship between the two constructs [31]. A study conducted on refugees found a moderately negative relationship between PTSD and PTG [41]. Two longitudinal studies conducted after natural disasters concluded that PTSD symptoms negatively affected PTG development [47,48]. In contrast, studies conducted among Syrian refugees living in Turkey have yielded mixed findings, with two studies reporting a positive association between PTSD and posttraumatic growth and one study identifying a curvilinear relationship between the two constructs. [15,35,49]. A curvilinear relationship between the two constructs was also found in a group of North Korean refugees [39]. A meta-analysis study found a linear significant relationship between PTSD and PTG. When certain factors were taken into account (age and type of trauma), a curvilinear relationship was found [50].

Studies reporting a curvilinear relationship have emphasized that moderate levels of PTSD symptoms are the strongest predictors of posttraumatic growth [35,51]. A consistent finding across the literature is that PTSD and PTG are related. However, factors beyond PTSD symptom severity may shape the direction and nature of this relationship. Variables such as the type of trauma experienced, age, time elapsed since displacement, and the use of different coping strategies may independently influence posttraumatic growth. Furthermore, cross-sectional studies may overlook the curvilinear relationship between the two constructs.

In the present study, dispositional mindfulness negatively affected PTSD and positively affected PTG, which is consistent with previous studies [52,53,54,55]. However, our further analysis shows that only high mindfulness scores have a positive effect on PTG. The importance of cognitive coping methods in understanding post-traumatic reactions has been emphasized [56]. Traumatic experiences can profoundly shake or even destroy a person’s fundamental assumptions about themselves and the world, such as the meaningfulness of life, the goodness of the world, and one’s self-worth [57]. Refugeehood is a process with a high probability of experiencing such traumatic events, perhaps repeatedly. Studies conducted with Syrian refugees have documented the prevalence of multiple traumatic experiences [58,59].

The development of posttraumatic growth is thought to require an initial disruption of an individual’s core beliefs and assumptive worldview [7]. It has been shown that those who experience greater disruption in their hypothetical world due to trauma can achieve higher PTG levels than those who do not experience such disruption [60]. However, the process of questioning or dismantling one’s assumptive worldview is often accompanied by significant psychological distress [61]. Calhoun and Tedeschi divided the cognitive processes that trigger PTG development into two categories: intrusive rumination and deliberate rumination. Intrusive rumination is the automatic and unexpected cognitive process that first emerges after trauma. They represent the person’s attempt to understand what has happened to them. Deliberate ruminations are thought processes in which the person consciously and constructively engages in cognitive processing. They develop after intrusive ruminations while trying to cope with the traumatic experience [61,62]. Through deliberate rumination, the person attempts to adapt to new life conditions and reconstruct core beliefs. A growing body of research has demonstrated that deliberate rumination is associated with PTG, while intrusive rumination is associated with PTSD [63,64]. In this context, the Mindfulness Meaning Theory (MMT) provides an important conceptual framework for understanding the transition from intrusive to deliberate rumination and the facilitation of PTG [65]. Meaning-making is one of the fundamental motivations of human existence. Catastrophic experiences such as trauma lead to uncertainty and a perceived loss of control [66]. People engage in efforts to establish new meaningful patterns to escape loss of control and uncertainty [67,68]. One of MMT’s hypotheses is that mindfulness encourages reappraisal and facilitates new meaning formation. Mindfulness enables a transition to a meta-cognitive level of awareness where new perspectives can be seen through the flexible use of attention and its removal from a narrow scope. This transition provides an opportunity to directly engage with and observe one’s thoughts and feelings. Increased attention to the emotional and cognitive effects of traumatic experiences leads to distancing from the experience, reappraisal of the event, and a decrease in avoidance behaviors. As a result, the transition from intrusive rumination to deliberate rumination, which is key to PTG, becomes easier [56].

It is also necessary to include the views of Dabrowski, who made significant contributions to explaining the development. The general approach of psychological therapy is to alleviate distress, anxiety, and crises, and restore stability. However, Dąbrowski’s theory of positive disintegration offers an alternative view. According to this theory, periods of depression, anxiety and crisis are viewed as essential components of personal growth.

This developmental process involves ‘loosening’, ‘splitting’, or ‘smashing’ the old reality. Positive disintegration generally refers to the transition of mental functions from lower to higher, broader, and richer levels. This transition requires the restructuring of mental functions [69].

Another focus of our research was to examine the relationship between mindfulness and the subdomains of PTG. According to our research findings, mindfulness was not effective in the Spiritual Change domain, while the strongest effect was found in the other four domains, with relating to others being the most effective. These results indicate that mindfulness is quite important in PTG development, although its effect sizes vary. In contrast, Redekop et al. reported weak and non-significant associations between mindfulness levels, as measured by the MAAS, and the non-spiritual domains of posttraumatic growth [70], highlighting potential contextual or sample-related differences across studies.

According to Tedeschi and Blevins, it has been suggested that similar growth outcomes should not be expected across all domains of PTG and that different developmental levels may occur over time. According to MMT, while mindfulness facilitates PTG by triggering different cognitive mechanisms, growth may occur in different domains depending on different traumatic and negative life experiences [71].

There is evidence suggesting that engagement in contemplative practices is associated with higher levels of PTG [65]. Notably, the Spiritual Change domain appears to be particularly influenced by such practices. In a study conducted by Hanley et al., the Spiritual Change PTG dimension was found to be significantly higher in contemplative practitioners compared to non-practitioners [53]. These findings suggest that dispositional mindfulness and mindfulness cultivated through contemplative practices may represent distinct processes that are differentially associated with specific domains of PTG. In this context, the role of contemplative practices in facilitating spiritual transformation is further supported by the available evidence.

Current evidence suggests that the relationship between mindfulness and PTG is complex and may involve multiple mediating factors. Future research should further examine which specific components of mindfulness are associated with different domains of PTG, as well as how these relationships may vary across different types of trauma. In addition, longitudinal studies investigating changes in this relationship over time and identifying potential influencing factors would provide valuable insights.

## 5. Limitations

This study has several limitations that should be acknowledged. First, data were collected from refugees residing in a single geographic location (Adana) and participation was based on voluntary sampling, which may limit the generalizability of the findings. In addition, the cross-sectional design and reliance on self-report measures necessitate caution when interpreting causal relationships among the study variables.

Furthermore, mindfulness was assessed using the MAAS, a unidimensional measure, which does not capture the multifaceted nature of mindfulness. Consequently, the relationships between specific mindfulness facets and posttraumatic growth could not be examined. These limitations should be considered when interpreting the results.

Despite these constraints, given the limited empirical evidence on the relationships among posttraumatic growth, PTSD, and mindfulness within refugee populations living in Turkey, the findings of the present study make an important contribution to the existing literature.

## 6. Conclusions

The refugee experience is a challenging process that can involve traumatic and negative life events. It can encompass various negative aspects before, during, and after migration. Previous research in this field has focused on the negative outcomes of the process. However, the refugee experience does not always have to be associated with negative psychological outcomes. Our research focused on the level of posttraumatic growth (PTG), which reflects positive change, and related factors and documented that mindfulness is an important factor in explaining PTG development. Identifying the factors associated with PTG and contributing to its development can contribute to a better understanding of the refugee population, which has a very challenging life experience, and to the development of better support systems.

## Figures and Tables

**Table 1:** Baseline statistics.

Variables	Statistics
**Age (mean ± sd)**	36.9 ± 10.4
**Gender (n (%))**	
Female	113 (47.1)
Male	127 (52.9)
**Marital status (n (%))**	
Married	212 (88.3)
Single	28 (11.7)
**Number of children (median (Q1** **–Q3))**	3 (2–4)
**Education duration (mean ± sd)**	6.5 ± 5.3
**Living arrangements (n (%))**	
Single	8 (0.3)
Family	232 (96.7)
**Household (median (Q1** **–Q3))**	5 (4–7)
**Relatives in Syria (n (%))**	
No	116 (48.3)
Yes	124 (51.7)
**Steady job (n (%))**	
No	107 (44.5)
Yes	133 (55.5)
**Income (n (%))**	
<Min. wage (USD 126)	74 (30.8)
>Min. wage (USD 126)	166 (69.2)
**Ongoing support (n (%))**	
No	82 (34.2)
Yes	158 (65.8)
**Conflict exposure (n (%))**	
No	170 (70.8)
Yes	70 (29.2)
**Functional Turkish (n (%))**	
No	96 (40.0)
Yes	144 (60.0)
**PCL (mean ± sd)**	25.0 ± 12.7
**PTGI (mean ± sd)**	67.9 ± 14.7
**MAAS (mean ± sd)**	74.0 ± 11.0

PCL—PTSD Checklist, Diagnostic and Statistical Manual of Mental Disorders; PTGI—Posttraumatic Growth Inventory; MAAS—Mindful Attention Awareness Scale.

**Table 2:** Comparison according to scale scores.

Variables	PCL	*p*	PTGI	*p*	MAAS	*p*
**Gender**						
Female	25.4 ± 12.6	0.688	70.4 ± 14.6	0.011	73.3 ± 10.9	0.29
Male	24.7 ± 12.8		65.6 ± 14.5		74.8 ± 11	
**Income**						
<Min Wage (USD 126)	31.5 ± 14.1	<0.001	56.1 ± 13.8	<0.001	68.2 ± 10.7	<0.001
>Min Wage (USD 126)	22.2 ± 10.9		73 ± 11.9		76.6 ± 10.1	
**Marital status**						
Married	24 (15–34)	0.045	68 (58–78)	0.928	75 (67–82)	0.92
Single	17 (12.5–26)		71.5 (55.5–78.5)		76 (64–82.5)	
**Relatives in Syria**						
No	24.2 ± 11.9	0.338	67.7 ± 13.2	0.872	74.6 ± 10.2	0.486
Yes	25.8 ± 13.4		68 ± 16.1		73.6 ± 11.7	
**Steady job**						
No	23 ± 11.5	0.026	68 ± 13.8	0.913	74.9 ± 10.1	0.257
Yes	26.6 ± 13.3		67.8 ± 15.5		73.3 ± 11.6	
**Ongoing support**						
No	25.7 ± 15	0.552	65.9 ± 16.7	0.147	75 ± 11.8	0.354
Yes	24.7 ± 11.3		68.8 ± 13.5		73.6 ± 10.5	

PCL—PTSD Checklist, Diagnostic and Statistical Manual of Mental Disorders; PTGI—Posttraumatic Growth Inventory; MAAS—Mindful Attention Awareness Scale.

**Table 3:** Path analysis for scales and significant variables.

Variables (i <-- j)	Unst. Weights	*p*	St. Weights	St. Indirect Effects	Ind.Se	Ind.CI	St. Total Effects
PTG <---- PTSD	−0.334	<0.001	−0.291	−0.254	0.069	−0.421–0.151	−0.545
PTG <---- Mindfulness	0.501	<0.001	0.378	-	-	-	0.378
Mindfulness <---- PTSD	−0.583	<0.001	−0.674	-	-	-	−0.674
PTSD <---- Edu.	−0.399	0.01	−0.165	-	-	-	−0.165
PTG <---- Age	−0.166	0.015	−0.118	-	-	-	−0.118
PTG <---- Edu	0.453	<0.001	0.163	0.05	0.046	0.008–0.259	0.253

Unst.: Unstandardized; St: Standardized; Edu: Education Duration; Ind: Indirect; Se: Standard Error; CI: Confidence Interval.

**Table 4:** Path analysis for PTG subscales.

Variables (i <-- j)	Unst. Weights	*p*	Correlations	St. Weights
PTG RO <---- PTSD	−0.112	0.028	−0.322	−0.179
PTG NP <---- PTSD	−0.078	0.013	−0.436	−0.186
PTG PS <---- PTSD	−0.059	0.004	−0.416	−0.22
PTG SC <---- PTSD	−0.043	0.001	−0.338	−0.263
PTG AL <---- PTSD	−0.054	0.016	−0.391	−0.187
PTG RO <---- Mindfulness	0.156	0.009	0.334	0.214
PTG NP <---- Mindfulness	0.18	<0.001	0.497	0.372
PTG PS <---- Mindfulness	0.091	<0.001	0.44	0.292
PTG SC <---- Mindfulness	0.021	0.176	0.287	0.111
PTG AL <---- Mindfulness	0.101	<0.001	0.429	0.303

Unst.: Unstandardized; St: Standardized. RO: Relating to Others; NP: New Possibilities; PS: Personal Strength; SC: Spiritual Change; AL: Appreciation of Life.

## Data Availability

The data presented in this study are not publicly available due to privacy considerations. Data may be available from the corresponding author on reasonable request.

## Abbreviations

The following abbreviations are used in this manuscript:

PTG	Posttraumatic Growth
PTGI	Posttraumatic Growth Inventory
PTSD	Posttraumatic Stress Disorder
MAAS	Mindful Attention Awareness Scale
PCL-5	Posttraumatic Stress Disorder Checklist for the Diagnostic and Statistical Manual of Mental Disorders, Fifth Edition
RO	Relating to Others
NP	New Possibilities
PS	Personal Strength
SC	Spiritual Change
AL	Appreciation of Life
